# Supplementary material for: CHEK1 is a synthetic lethal interactor of FBXO7 in colonic epithelial cells
Source: Mol Ther Oncol. 2025 Aug 5;33(3):201028. doi: 10.1016/j.omton.2025.201028 (PMC12390932; doi:10.1016/j.omton.2025.201028)
Supplement: Document S1. Figure S1 and Tables S1–S8 [file mmc1.pdf]

OMTON, Volume *33*

Supplemental information

CHEK1 is a synthetic lethal interactor of *FBXO7* in colonic epithelial cells

Tooba Razi, Ally C. Farrell, Rubi Campos Gudiño, Nicole M. Neudorf, Zelda Lichtensztejn, and Kirk J. McManus

SUPPLEMENTAL MATERIAL

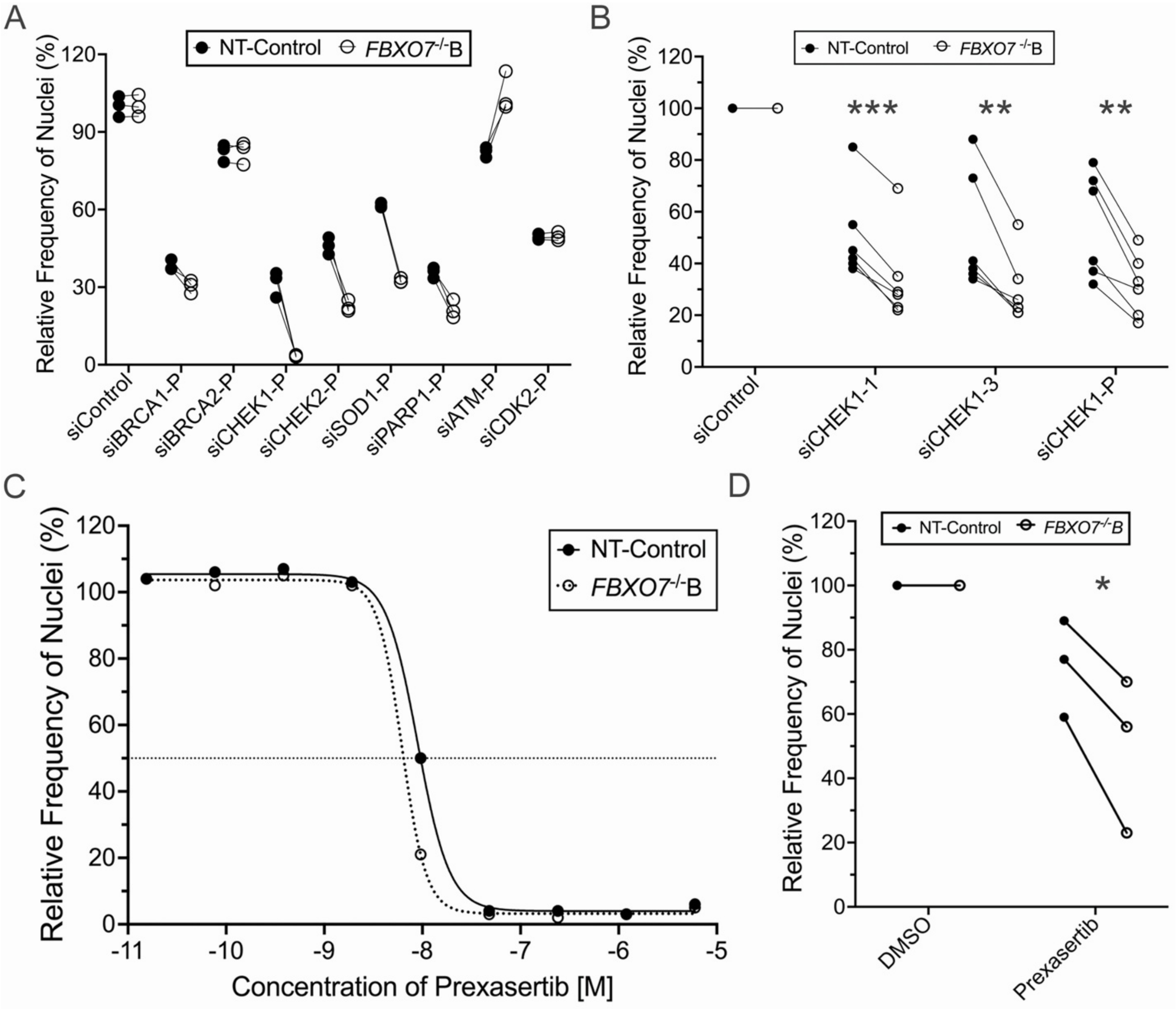

Figure S1: *FBXO7*$^{-/-}$B cells are Hypersensitive to CHEK1 Silencing and Inhibition.
(**A**) Pilot screen reveals *FBXO7*$^{-/-}$B cells are hypersensitive to *CHEK1*, *CHEK2*, *SOD1* and *PARP1* silencing (n = 3). **(B)** QuantIM identifies statistically significant decreases in the relative frequency of *FBXO7*$^{-/-}$B nuclei (cells) relative to NT-Control following *CHEK1* silencing (n = 6, multiple paired t-tests, with Benjamini, Krieger and Yekutieli correction; FDR = 5%; **, q-value < 0.01; ***, q-value < 0.001; Table S2). **(C)** Dose response curve reveals *FBXO7*$^{-/-}$B cells are hypersensitive to Prexasertib treatments (5-fold serial dilution) relative to NT-Control. Mean values are presented normalized to the corresponding DMSO control (n = 6). **(D)** QuantIM identifies a statistically significant decrease in the relative frequency of *FBXO7*$^{-/-}$B nuclei (cells) relative to NT-Control cells following Prexasertib (6.0μM) treatment (n = 3, multiple paired t-tests, with Benjamini, Krieger and Yekutieli correction; FDR = 5%; *, q-value < 0.05; Table S4).

Table S1. ***CHEK1*** **Silencing Induces Statistically Significant Decreases in the Number of** ***FBXO7*** $^{-/-}$**A Cells**[A]**.**

	p-value (×10^{-2})	Mean (NT-Cntl)	Mean (*FBXO7*$^{-/-}$)	Diff[B]	SE[C]	t-ratio	df[D]	q-value[E] (×10^{-2})
**siControl**		100.0	100.0	0.00	0.00			
**siCHEK1-1**	2.1221	52.7	29.5	23.2	7.00	3.31	5	2.526
**siCHEK1-3**	2.4056	52.5	29.8	22.7	7.09	3.20	5	2.526
**siCHEK1-P**	1.0794	51.2	25.5	25.7	6.49	4.00	5	2.526

[A]Multiple paired t-tests
[B]Difference, Diff
[C]Standard error, SE
[D]Degrees of freedom, df
[E]two-stage step-up Benjamini, Krieger and Yekutieli multiple comparison correction with Q = 5%

Table S2. ***CHEK1*** **Silencing Induces Statistically Significant Decreases in the Number of** ***FBXO7*** $^{-/-}$**B Cells**[A]**.**

	p-value (×10^{-2})	Mean (NT-Cntl)	Mean (*FBXO7*$^{-/-}$)	Diff[B]	SE[C]	t-ratio	df[D]	q-value[E] (×10^{-2})
**siControl**		100.0	100.0	0.00	0.00			
**siCHEK1-1**	0.0108	50.8	34.33	16.5	1.50	11.0	5	0.0340
**siCHEK1-3**	0.6783	51.7	30.00	21.7	4.88	4.44	5	0.6851
**siCHEK1-P**	0.3384	54.8	31.50	23.3	4.46	2.23	5	0.5127

[A]Multiple paired t-tests
[B]Difference, Diff
[C]Standard error, SE
[D]Degrees of freedom, df
[E]two-stage step-up Benjamini, Krieger and Yekutieli multiple comparison correction with Q = 5%

Table S3. Prexasertib Induces a Significant Decreases in the Number of ***FBXO7*** $^{-/-}$**A Cells.**

Condition	p-value (×10^{-2})	Mean (NT-Cntl)	Mean (*FBXO7*$^{-/-}$)	Diff[B]	SE[C]	t-ratio	df[D]	q-value[E] (×10^{-2})
DMSO		100.0	100.0	0.00	0.00			
Prexasertib	4.0826	72.7	40.33	32.3	6.74	4.80	2	4.286

[A]Multiple paired t-tests
[B]Difference, Diff
[C]Standard error, SE
[D]Degrees of freedom, df
[E]two-stage step-up Benjamini, Krieger and Yekutieli multiple comparison correction with Q = 5%

Table S4. Prexasertib Induces a Significant Decreases in the Number of *FBXO7*$^{-/-}$B Cells.

Condition	p-value (×10^{-2})	Mean (NT-Cntl)	Mean (*FBXO7*$^{-/-}$)	Diff[B]	SE[C]	t-ratio	df[D]	q-value[E] (×10^{-2})
DMSO		100.0	100.0	0.00	0.00			
Prexasertib	4.2034	75.00	49.7	25.33	5.36	4.72	2	4.414

[A]Multiple paired t-tests
[B]Difference, Diff
[C]Standard error, SE
[D]Degrees of freedom, df
[E]two-stage step-up Benjamini, Krieger and Yekutieli multiple comparison correction with Q = 5%

Table S5. Prexasertib Induces Preferential Increases in γ-H2AX Foci in *FBXO7*$^{-/-}$A Cells.

Cell Type	Treatment	N[A]	Mean ± SD[B]	Fold Increase[C]
NT-Control	DMSO	750	0.2067 ± 1.115	NA
NT-Control	Prexasertib	693	3.221 ± 16.03	15.6
FBXO7$^{-/-}$A	DMSO	596	0.4279 ± 1.462	NA
FBXO7$^{-/-}$A	Prexasertib	552	17.81 ± 36.75	41.6

[A]Number (N)
[B]Standard Deviation (SD)
[C]Relative to the corresponding DMSO-treated control of the identical cell line; Not applicable (NA)

Table S6. Dunn's Multicomparison Tests Reveals Statistically Significant Increases γ-H2AX Foci Following Prexasertib Treatments.

	NT-Control (Prexasertib)	*FBXO7*$^{-/-}$A (DMSO)	*FBOX7*$^{-/-}$A (Prexasertib)
NT-Control (DMSO)	****[A]	****	****
NT-Control (Prexasertib)	****	ns	****
FBXO7$^{-/-}$A (DMSO)			****

[A]Statistical Significance; p-value > 0.05 (ns); p-value <0.0001 (****)

Table S7. Prexasertib Induces Preferential Increases in Cleaved Caspase 3 Total Signal Intensities in *FBXO7*$^{-/-}$A Cells.

Cell Type	Treatment	N[A]	Mean ± SD[B]	Fold Increase[C]
NT-Control	DMSO	799	4922 ± 1249	NA
NT-Control	Prexasertib	611	8474 ± 2690	1.7
FBXO7$^{-/-}$A	DMSO	637	7439 ± 1920	NA
FBXO7$^{-/-}$A	Prexasertib	574	19012 ± 6020	2.6

[A]Number (N)
[B]Standard Deviation (SD)
[C]Relative to the corresponding DMSO-treated control of the identical cell line; Not applicable (NA)

Table S8. Dunn's Multicomparison Tests Reveals Statistically Significant Increases in Cleaved Caspase 3 Total Signal Intensities Following Prexasertib Treatments.

	NT-Control (Prexasertib)	*FBXO7*$^{-/-}$A (DMSO)	*FBOX7*$^{-/-}$A (Prexasertib)
NT-Control (DMSO)	****[A]	****	****
NT-Control (Prexasertib)		****	****
FBXO7$^{-/-}$A (DMSO)			****

[A]Statistical Significance; p-value <0.0001 (****)
