# Supplementary material for: CHEK1 is a synthetic lethal interactor of FBXO7 in colonic epithelial cells
Source: Mol Ther Oncol. 2025 Aug 5;33(3):201028. doi: 10.1016/j.omton.2025.201028 (PMC12390932; doi:10.1016/j.omton.2025.201028)
Supplement: Document S2. Article plus supplemental information [file mmc2.pdf]

Molecular Therapy
Oncology
Original Article

CHEK1 is a synthetic lethal interactor of *FBXO7* in colonic epithelial cells

Tooba Razi,[1,2] Ally C. Farrell,[1,2] Rubi Campos Gudiño,[1,2] Nicole M. Neudorf,[1,2] Zelda Lichtensztejn,[1,2] and Kirk J. McManus[1,2]

[1]Department of Biochemistry and Medical Genetics, University of Manitoba, Winnipeg, MB, Canada; [2]Paul Albrechtsen Research Institute CancerCare Manitoba, Winnipeg, MB, Canada

Colorectal cancer (CRC) remains a leading cause of cancer mortality worldwide, with chromosome instability (CIN) present in approximately 85% of cases and associated with poor prognosis. Reduced expression of *FBXO7*, a component of the SKP1-CUL1-F-box (SCF) E3 ubiquitin ligase complex, occurs in about one-third of CRCs and correlates with CIN, positioning *FBXO7* as a potential therapeutic target. This study employed bioinformatics analyses, small interfering RNA (siRNA) screening, small molecule inhibition, and quantitative imaging (QuantIM) microscopy to identify synthetic lethal interactors of *FBXO7*. Shallow deletions of *FBXO7* in CRC patient samples was found to associate with decreased gene expression and adverse clinical outcomes. Targeted silencing or pharmacological inhibition of CHEK1 using Prexasertib significantly reduced proliferation in *FBXO7*-deficient cells. Mechanistic studies revealed that Prexasertib treatment increased DNA double-strand breaks and apoptosis specifically in *FBXO7*-deficient cells. Furthermore, combining Prexasertib with 5-fluorouracil, a standard chemotherapeutic agent, produced a synergistic killing effect. These findings establish a novel synthetic lethal relationship between *FBXO7* and *CHEK1*, suggesting that CHEK1 inhibition may provide a targeted therapeutic strategy for CRC patients with *FBXO7* deficiencies, and highlighting the broader potential of exploiting SCF complex alterations in CRC therapy.

INTRODUCTION

Colorectal cancer (CRC) remains a significant global health burden. In 2022, CRC was the third most diagnosed cancer, with ~1.9 million new cases, and the second leading cause of cancer-related deaths, accounting for around 930,000 fatalities.[1] Alarmingly, the International Agency for Research on Cancer projects that by 2040, CRC incidence will rise to 3.2 million new cases annually, with 1.6 million deaths.[2] Despite advances in early detection and screening, more than half of all CRC cases are still diagnosed at advanced stages (III and IV),[3,4] when chemotherapy and targeted therapies are often the only viable treatment options. These statistics underscore a pressing need for more effective precision medicine strategies to improve outcomes for CRC patients. Exploiting the aberrant genetics that drive CRC development and progression represents a promising avenue, as tailoring treatments to individual genetic profiles may ultimately improve both survival and quality of life.

The molecular determinants (i.e., the aberrant genes, proteins, and pathways) underlying CRC pathogenesis are critical not only for prognostication but also for the development of innovative therapeutic approaches. Targeting these molecular drivers has the potential to enhance treatment specificity and minimize adverse effects. Chromosome instability (CIN), the predominant form of genomic instability in CRC, is observed in ~85% of cases.[5] CIN, defined as an increased rate of whole chromosome or large chromosomal segment gains and losses, drives both genetic and cellular heterogeneity.[5–7] CIN can be subdivided into numerical (aneuploidy) and structural (amplifications, deletions, duplications, translocations, inversions) categories. It is implicated in all aspects of cancer biology, including tumor initiation,[8–12] inter- and intra-tumoral heterogeneity,[13,14] metastasis,[15,16] and the acquisition of drug resistance,[17,18] and is frequently associated with poor patient outcomes.[19–21] Despite these associations, the molecular determinants underlying CIN in CRC remain poorly understood. Recent work from our group has demonstrated that copy number loss and reduced expression of key SKP1-CUL1-F-box (SCF) complex genes induce CIN,[8–12] identifying these genes as potentially exploitable therapeutic targets.

The SCF complex is an E3 ubiquitin ligase responsible for polyubiquitylation and subsequent degradation of target proteins by the 26S proteasome.[22] It consists of three invariable core members (SKP1, CUL1, RBX1) and one of 69 variable F-box proteins that confer substrate specificity. Among these, FBXO7 is of particular interest as gene copy number loss occurs in approximately 33% of CRC cases (~608,000 individuals annually) and is associated with reduced expression and worse patient outcomes.[11,23,24] Conceptually, reduced *FBXO7* expression impairs the ability of the SCF complex to regulate key substrates involved in cell-cycle progression and genome maintenance, leading to CIN and cellular transformation. In CRC, *FBXO7*

Received 1 May 2025; accepted 30 July 2025;
https://doi.org/10.1016/j.omton.2025.201028.

Correspondence: Kirk J. McManus, Paul Albrechtsen Research Institute CancerCare Manitoba, ON6038–675 McDermot Avenue, Winnipeg, MB R3E 0V9, Canada.
E-mail: kirk.mcmanus@umanitoba.ca

© 2025 The Author(s). Published by Elsevier Inc. on behalf of The American Society of Gene and Cell Therapy. This is an open access article under the CC BY license (http://creativecommons.org/licenses/by/4.0/).

copy number loss is not only prevalent but is significantly associated with higher levels of CIN, aneuploidy, and poor patient outcomes,[11,23,24] suggesting a pathogenic role in disease development and progression. Our recent study further implicates *FBXO7* deficiency in the disruption of mitotic fidelity and DNA damage response pathways, highlighting its potential importance as a tumor suppressor in colonic epithelial cell contexts.[11] More specifically, we determined that *FBXO7* silencing induces CIN in both non-malignant and malignant human colonic epithelial cells, and that CRISPR-Cas9-generated *FBXO7*$^{+/-}$ and *FBXO7*$^{-/-}$ clones exhibit dynamic CIN phenotypes and cellular transformation over time.[11] Together, these data suggest that reduced *FBXO7* expression contributes to CRC development and progression, rendering it an attractive candidate for therapeutic exploitation through a synthetic lethal (SL) approach.

Synthetic lethality describes a scenario in which the combination of two independent gene deficiencies is lethal, while each deficiency alone is tolerated (reviewed in O'Neil et al.[25]). This strategy has been successfully translated into the clinic, as seen with PARP inhibitors in *BRCA1/2*-defective cancers.[26,27] Intuitively, targeting the core SCF complex members is unlikely to provide the specificity required for precision medicine due to their broad regulatory roles and potential off-target effects. In contrast, exploiting defects in a single F-box gene, like *FBXO7*, may restrict cytotoxicity to cancer cells while sparing normal tissues.

In this study, we identify and characterize a novel SL interaction between *FBXO7* and *CHEK1*. CHEK1 is a serine/threonine kinase that plays a pivotal role in the DNA damage response, particularly in mediating cell-cycle arrest in response to replication stress and DNA double-strand breaks. By halting cell-cycle progression, CHEK1 allows time for DNA repair and thus preserves genomic integrity.[28,29] Tumor cells with underlying defects in genome maintenance, such as FBXO7 deficiency, are hypothesized to be particularly reliant on CHEK1 function for survival, making CHEK1 inhibition an attractive SL strategy in this context. Using two independent *FBXO7*$^{-/-}$ human colonic epithelial cell models and matched control,[11] we show that *CHEK1* silencing via small interfering RNA (siRNA) preferentially reduces cell numbers in *FBXO7*-deficient cells. Pharmacological inhibition of CHEK1 with Prexasertib recapitulates this effect, leading to increased DNA double-strand breaks and apoptosis, as indicated by increases in γ-H2AX foci and cleaved caspase-3 signal intensities. Additionally, we demonstrate that Prexasertib synergizes with 5-fluorouracil (5-FU), a standard chemotherapeutic agent in CRC. Collectively, our findings suggest that CHEK1 inhibition represents a promising therapeutic strategy for CRC patients harboring *FBXO7* defects, and warrants further pre-clinical evaluation, particularly in combination with established chemotherapies.

RESULTS

FBXO7 is frequently lost in cancer and is associated with genome instability and poor patient outcomes in CRC

Prior to identifying novel SL interactors (i.e., drug targets) of *FBXO7*, we first employed bio-informatic approaches to assess the prevalence and clinical impact of *FBXO7* copy number alterations. Using publicly available patient datasets from The Cancer Genome Atlas (TCGA), we determined that copy number losses are more prevalent than gains in nine of 10 solid tumor types (Figure 1A). We further noted that shallow deletions (i.e., heterozygous losses, or loss of a single allele) occur in all 10 cancer types and range from 7.6% in kidney cancer to 43.5% in breast cancer, while deep deletions (i.e., homozygous losses, or loss of both alleles) are rare and occur in <0.5% of cases in any cancer type (Figure 1A). Given our recent experience with members of the SCF complex and CRC,[8–12] we focused our attention on the clinical impacts of *FBXO7* copy number alterations in CRC. Overall, *FBXO7* copy number losses are more prevalent than gains, as shallow and deep deletions occur in 32.5% (169 of 526 cases) and <0.2% (1 of 526) of CRC cases, respectively, while gains and amplifications only occur in 4% (19 of 526) and <0.2% (1 of 526) of cases; mutations occur in ∼1% (7 of 526) of cases (Figure 1B). Furthermore, CRCs with shallow deletions exhibit a significant reduction in mRNA expression relative to diploid cases (Figure 1C). Unfortunately, a comprehensive immunohistochemical evaluation of FBXO7 (i.e., at the protein level) has never been conducted in CRC. In agreement with our previous study demonstrating that reduced *FBXO7* expression induced CIN,[11] CRC cases with shallow deletions also exhibit significant increases in genome instability, including the fraction of the genome altered, aneuploidy score, and tumor break load (Figure 1D). Briefly, the fraction of the genome altered refers to the percentage of the genome affected by copy number alterations (gains and losses),[30] while aneuploidy scores are a measure of copy number alterations at the chromosome arm level,[31] and tumor break load is a measure used to quantify chromosomal structural variants.[32] Finally, we determined that shallow deletions are associated with worse CRC patient outcomes (Figure 1E), including overall, progression free, and disease-specific survival. Collectively, these clinical data, coupled with our previous findings that reduced *FBXO7* induces CIN that promotes cellular transformation,[11] are consistent with *FBXO7* copy number losses being a pathogenic contributor in CRC and identify *FBXO7* as an ideal target to exploit using an SL paradigm.

FBXO7 and *CHEK1* are SL interactors in human colonic epithelial cells

Building on the clinical association between *FBXO7* copy number loss and CRC, we investigated SL interactors that could selectively target and kill *FBXO7*-deficient cells. Guided by our previous experience,[33–36] reagent availability, and the therapeutic potential of targeting the DNA damage response (DDR), notably through PARP inhibition,[26,27] we performed a small SL screen involving eight key DDR members: *BRCA1*, *BRCA2*, *CHEK1*, *CHEK2*, *SOD1*, *PARP1*, *ATM*, and *CDK2*. However, we first assessed FBOX7 abundance and reconfirmed that the *FBXO7*$^{-/-}$A clone does not express FBXO7 (Figure 2A).[11] Next, each DDR gene was independently silenced in both the NT-Control and the *FBXO7*$^{-/-}$A clones using our established protocol.[37] Silencing of *CHEK1*, *CHEK2*, and *SOD1* resulted in consistent reductions in *FBXO7*$^{-/-}$A cell numbers relative to NT-Control (Figure 2B), with analogous effects also observed within

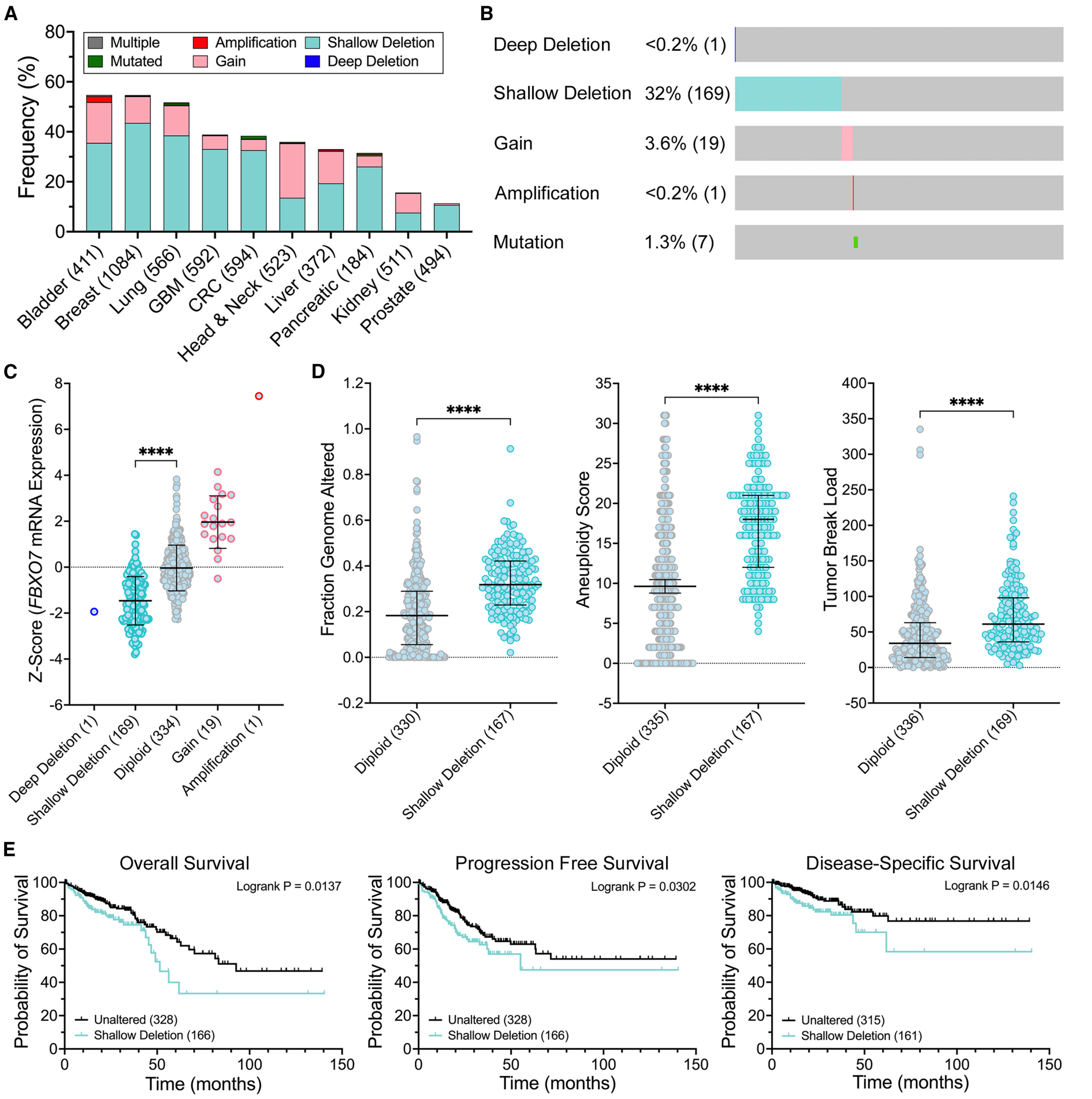

Figure 1. Prevalence and clinical implications for *FBXO7* copy number losses in cancer

(A) Bar graph presenting the frequency of *FBXO7* alterations including copy number losses (deep [homozygous] and shallow [heterozygous] deletions), gains (gains and amplifications), and mutations in 10 solid cancer types.[23,24] Total number of cases indicated in brackets. (B) Frequency of *FBXO7* alterations and mutations in CRC (number of cases/526 total cases). Gray shading represents unaltered cases. (C) CRC cases with *FBXO7* shallow deletions have significantly reduced mRNA expression relative to diploid cases (Mann-Whitney test; ****p value <0.0001; bars present mean ± SD). (D) Dot plots reveal CRC cases with shallow deletions exhibit significant increases in genome instability, including the fraction of the genome altered (left), aneuploidy scores (middle), and tumor break load (right) (Wilcoxon test with Benjamini-Hochberg FDR; ****q value <0.0001; bars identify median and interquartile ranges). (E) Kaplan-Meier curves reveal significantly worse overall (left), progression free (middle), and disease-specific (right) survival for CRC cases with shallow deletions relative to unaltered cases (log rank $p < 0.05$ is significant).

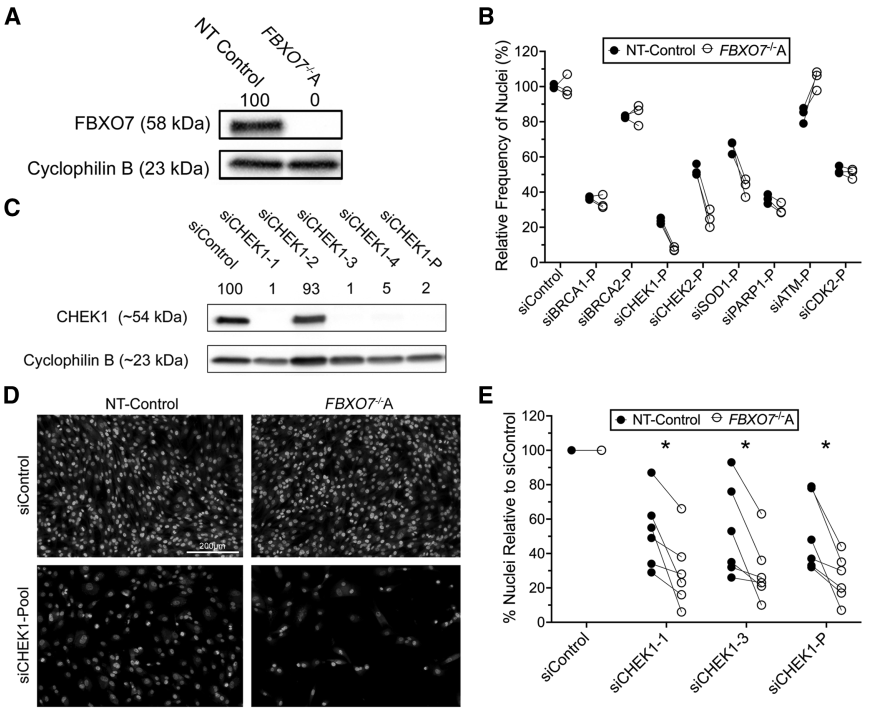

Figure 2. *CHEK1* is a putative SL interactor of *FBXO7*

(A) Western blot presenting FBXO7 abundance in NT-Control and $FBXO7^{-/-}$A clones, with Cyclophilin B serving as a loading control. FBXO7 abundance is normalized to the corresponding loading control and is presented relative to the NT-Control, which is set to 100%. (B) Silencing-based screen of eight candidate DDR genes reveals $FBXO7^{-/-}$A cells are hypersensitive to *CHEK1*, *CHEK2*, and *SOD1* silencing (n = 3). (C) Semi-quantitative western blot demonstrating silencing of both individual and pooled siRNAs targeting *CHEK1*, with siCHEK1-1, -3, and -P selected for subsequent study. (D) Representative low-resolution image (10×) showing a visual decrease in cellularity following *CHEK1* silencing in $FBXO7^{-/-}$A cells relative to NT-Control. Scale bar represents 200 μm. (E) Quantitative imaging microscopy (QuantIM) identifies statistically significant decreases in the relative frequency of nuclei (cells) remaining in $FBXO7^{-/-}$A compared with NT-Control clones following *CHEK1* silencing (n = 6, multiple paired t tests, with Benjamini, Krieger, and Yekutieli multi-comparison correction; FDR = 5%; *q value <0.05; Table S1).

the $FBXO7^{-/-}$B clone (Figure S1A). CHEK1 emerged as the lead candidate due to its central role in replication stress response, DDR, and cell-cycle checkpoint control,[28,38] whereas CHEK2 and SOD1 are primarily implicated in checkpoint signaling and oxidative stress management, respectively.[39] Efficient *CHEK1* silencing was confirmed (Figure 2C), after which targeted silencing was performed using individual (siCHEK1-1, and -3) and pooled siRNAs. Consistent with an SL interaction, CHEK1 depletion led to both visually apparent (Figure 2D) and statistically significant reductions in $FBXO7^{-/-}$A cell numbers relative to NT-Control (Figure 2E; Table S1), with similar results observed for the $FBXO7^{-/-}$B clone (Figure S1B; Table S2). The consistent reduction in *FBXO7*-deficient cell numbers within each clone reveal a previously unrecognized SL relationship between *FBXO7* and *CHEK1*, establishing CHEK1 as a candidate therapeutic target warranting further investigation.

$FBXO7^{-/-}$ cells are hypersensitive to Prexasertib treatments

To evaluate whether pharmacological CHEK1 inhibition replicates the effects of genetic *CHEK1* silencing, we treated NT-Control and $FBXO7^{-/-}$A clones with increasing Prexasertib concentrations. Dose-response analyses reveal enhanced sensitivity in $FBXO7^{-/-}$A cells (half maximal effective concentration [EC_{50}] = 5.83 nM) compared with NT-Control (EC_{50} = 9.03 nM) (Figure 3A). This hypersensitivity phenotype also extended to the $FBXO7^{-/-}$B clones, which show comparable dose-dependent hypersensitivity and reductions in cell numbers (Figure S1C). At the selected 6-nM concentration, Prexasertib treatment significantly reduced $FBXO7^{-/-}$A (Figure 3B; Table S3) and $FBXO7^{-/-}$B (Figure S1D; Table S4) cell numbers relative to NT-Control. The consistent response across both $FBXO7^{-/-}$ clones confirms that *FBXO7* loss sensitized cells to CHEK1 inhibition, mirroring our earlier observations following *CHEK1* silencing. Collectively, these results demonstrate that pharmacological CHEK1 inhibition phenocopies genetic *CHEK1* silencing in *FBXO7*-deficient models. Moreover, the parallel responses in both $FBXO7^{-/-}$ clones strengthen the conclusion that *FBXO7* status determines cellular dependency on CHEK1 activity for survival.

Prexasertib treatments induce increases in γ-H2AX in $FBXO7^{-/-}$A cells

Having identified Prexasertib as a novel candidate drug in $FBXO7^{-/-}$ cells, we next sought to gain insight into the underlying mechanism giving rise to the reduction in cell numbers. Given that CHEK1 normally functions in replication stress,[28,38] we reasoned that Prexasertib treatments would promote replication defects, ultimately leading to increases in DNA double-strand breaks (DSBs) within interphase cells. To test this possibility, NT-Control and $FBXO7^{-/-}$A clones were treated with DMSO or Prexasertib (6.0 nM) for 48 h at which point cells were fixed, immunofluorescently labeled (γ-H2AX; surrogate marker of DSBs[40]), counterstained (Hoechst), and subjected to quantitative imaging microscopy (QuantIM) to quantify and statistically compare the number of γ-H2AX foci between conditions and cell lines. As shown in Figure 4, Prexasertib treatments induced significant increases in γ-H2AX foci in both the NT-Control and the $FBXO7^{-/-}$A clone; however, the increase was greatest within the $FBXO7^{-/-}$A clone. More specifically, Prexasertib treatments induced a 41.6-fold increase in mean γ-H2AX foci within the $FBXO7^{-/-}$A clone (Table S5), while only a 15.6-fold increase was

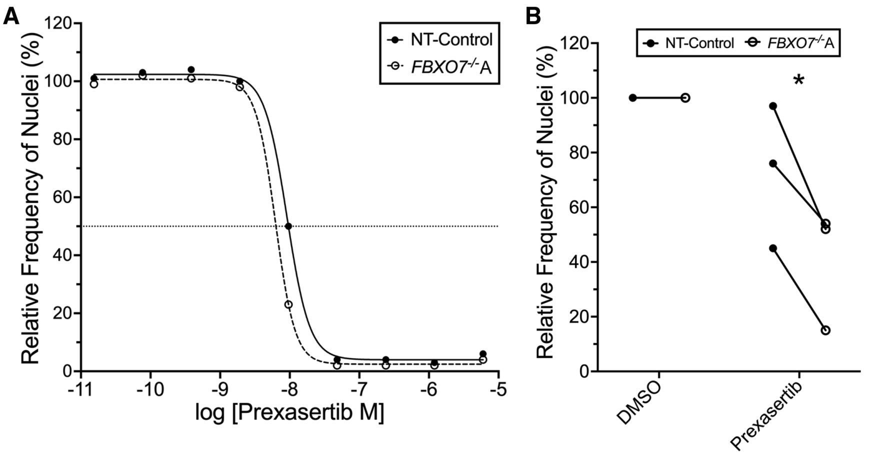

Figure 3. *FBXO7*$^{-/-}$ cells are hypersensitive to Prexasertib

(A) Representative dose-response curve (5-fold serial dilution) revealing *FBXO7*$^{-/-}$A cells are hypersensitive to Prexasertib (CHEK1 inhibitor) treatments relative to NT-Control (EC_{50} = 5.83 nM and 9.03 nM, respectively). Data are presented normalized to the corresponding DMSO control (n = 6). (B) Dot plots presenting the relative frequency of nuclei (cells) remaining relative to DMSO following treatment with Prexasertib (6.0 nM) within NT-Control and *FBXO7*$^{-/-}$A clones. Multiple paired t tests, with Benjamini, Krieger, and Yekutieli multi-comparison correction (FDR = 5%; *q value <0.05; Table S3) reveals a significant decrease in the relative frequency of nuclei (cells) within *FBXO7*$^{-/-}$A clones compared with NT-Control clones following Prexasertib treatment (n = 3).

observed within the NT-Control clone. Additionally, Kruskal-Wallis tests with Dunn's multiple comparison post-tests (Table S6) revealed statistically significant differences that were more pronounced within the *FBXO7*$^{-/-}$A clone treated with Prexasertib relative to the DMSO control. Collectively, these data show that while Prexasertib treatments induce increases in γ-H2AX foci in both cellular contexts, they are greatly enhanced within the *FBXO7*$^{-/-}$A cells, which is consistent with treatments inducing replication stress and DSBs that are preferentially enhanced with an *FBXO7*-deficient background.

Prexasertib induces increases in apoptosis in *FBXO7*$^{-/-}$ cells

Next, we investigated whether the Prexasertib-induced increases in DNA DSBs (γ-H2AX foci) lead to increases in apoptosis within the *FBXO7*-deficient cells. To assess this, we measured levels of cleaved caspase-3, a key indicator of apoptotic induction, that was purposefully selected as it is the convergence point for both the extrinsic and intrinsic apoptotic pathways.[41] Moreover, as the primary executioner caspase responsible for apoptosis, its cleaved form provides a reliable measure of apoptotic activity. Using a similar experimental design to that of the preceding section, cells were treated with DMSO or Prexasertib and permitted to grow for 48 h, at which point cells were fixed, immunofluorescently labeled (cleaved caspase-3), counterstained, and subjected to QuantIM to enable the quantification of cleaved caspase-3 total signal intensities within interphase nuclei. In agreement with the γ-H2AX data, Prexasertib induced significant increases in cleaved caspase-3 signal intensities within the NT-Control and *FBXO7*$^{-/-}$A (Figure 5; Table S7), with the largest (2.6-fold) increases occurring within the *FBXO7*$^{-/-}$A clone. Kruskal-Wallis tests with Dunn's multiple comparison post-tests (Table S8) revealed significant differences in cleaved caspase-3 signal intensities within both cell populations, with pronounced effects observed within the *FBXO7*$^{-/-}$A clone. Collectively, these findings are consistent with Prexasertib treatments inducing apoptosis preferentially within the *FBXO7*$^{-/-}$ cells.

Treatment with Prexasertib synergizes with 5-FU to enhance killing in *FBXO7*$^{-/-}$ cells

Recent advances in targeted therapies have highlighted the value of combination strategies to enhance anti-tumor efficacy, particularly in CRC, where resistance to single agents remains a significant challenge. Given the established clinical utility of 5-FU, a pyrimidine analog that disrupts thymidylate synthase and impairs DNA replication, repair, and transcription,[42–44] we sought to explore whether combining 5-FU with Prexasertib could yield synergistic effects in an *FBXO7*-deficient cellular context. To investigate this, NT-Control and *FBXO7*$^{-/-}$A clones were treated with varying concentrations of Prexasertib and 5-FU, both individually and in combination. Cell survival was quantified using QuantIM, and synergy was assessed via the Loewe Additivity model using Combenefit software.[45] As shown in Figure 6, Prexasertib and 5-FU produces a synergistic reduction in cell numbers within a defined concentration window (1.92 nM–9.6 nM Prexasertib with 28 μM 5-FU). Notably, at lower concentrations of 5-FU (1.1 μM–5.6 μM) combined with 1.92 nM Prexasertib, an antagonistic interaction was observed. These findings underscore the potential of rationally designed combination regimens to enhance therapeutic efficacy in CRC, while also emphasizing the importance of optimizing dosing strategies to maximize synergy and minimize antagonism. This approach aligns with current efforts in oncology to leverage combinatorial treatments for improved patient outcomes.

DISCUSSION

In this study, we identified *CHEK1* as a novel SL interactor of *FBXO7*, presenting a potential therapeutic target for *FBXO7*-deficient CRCs. Through a series of siRNA-based experiments and QuantIM, we demonstrated that *CHEK1* silencing significantly reduced the number of *FBXO7*$^{-/-}$A and *FBXO7*$^{-/-}$B cells relative to NT-Control. Subsequent chemogenetic tests revealed that Prexasertib could effectively mimic *CHEK1* silencing, preferentially decreasing *FBOX7*$^{-/-}$A and *FBXO7*$^{-/-}$B cell numbers. This reduction corresponded with increased γ-H2AX foci and cleaved caspase-3 levels,

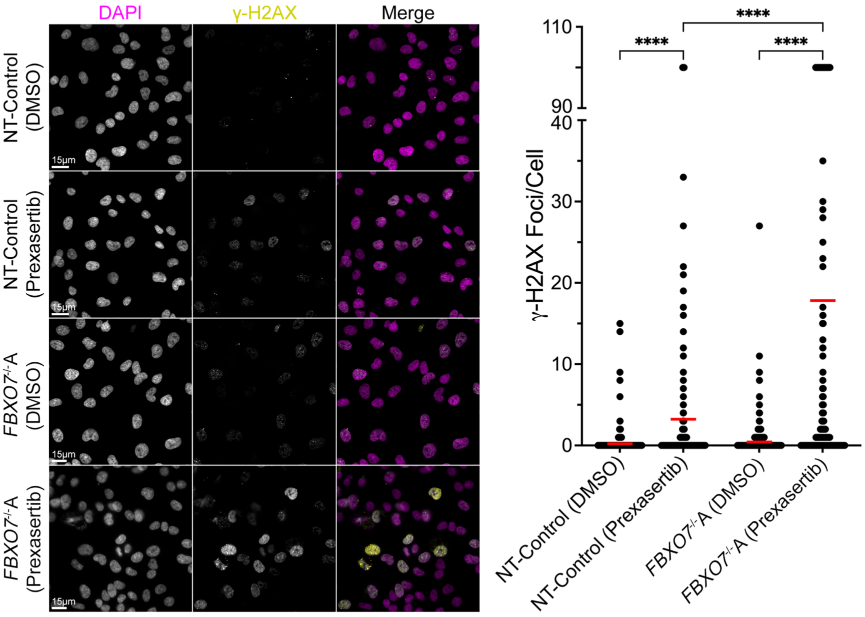

Figure 4. Prexasertib induces preferential increases in γ-H2AX foci in *FBXO7*$^{-/-}$A cells

Prexasertib (6.0 nM for 48 h) treatments induce visual (left) and statistically significant (right) increases in the number of γ-H2AX foci (i.e., DNA DSBs) within *FBXO7*$^{-/-}$A cells relative to DMSO-treated cells (Table S5). A Kruskal-Wallis test with Dunn's multiple comparison post-tests (****p value <0.0001; Table S6) were performed, with red lines identifying mean values (n = 1; >550 cells/condition).

indicating enhanced DNA DSBs and apoptosis, respectively. Furthermore, we observed a synergistic interaction between Prexasertib and 5-FU at specific concentrations, although an antagonistic effect was noted at lower 5-FU doses. While unexplored and speculative, the antagonistic effect observed at low doses of Prexasertib and 5-FU may be due to cell-cycle dynamics and checkpoint adaptation. Prexasertib is a CHEK1 inhibitor that abrogates the S and G2/M checkpoints, pushing cells through the cell cycle despite DNA damage. At low concentrations, Prexasertib may only partially inhibit CHEK1, allowing some checkpoint function to persist. This partial inhibition could enable cells to repair 5-FU-induced DNA damage more effectively, thereby reducing the cytotoxic synergy expected from full CHEK1 inhibition. This partial inhibition could enable cells to repair 5-FU-induced DNA damage more effectively, thereby reducing the cytotoxic synergy expected from full CHEK1 inhibition. Nevertheless, our findings establish CHEK1 as a promising drug target and Prexasertib as a lead candidate therapeutic agent for CRCs exhibiting *FBXO7* copy number losses, warranting further pre-clinical investigation.

Building upon our findings, it is important to consider the multifaceted roles of FBXO7 in cellular processes and their implications for cancer biology. FBXO7 exhibits both SCF-dependent and

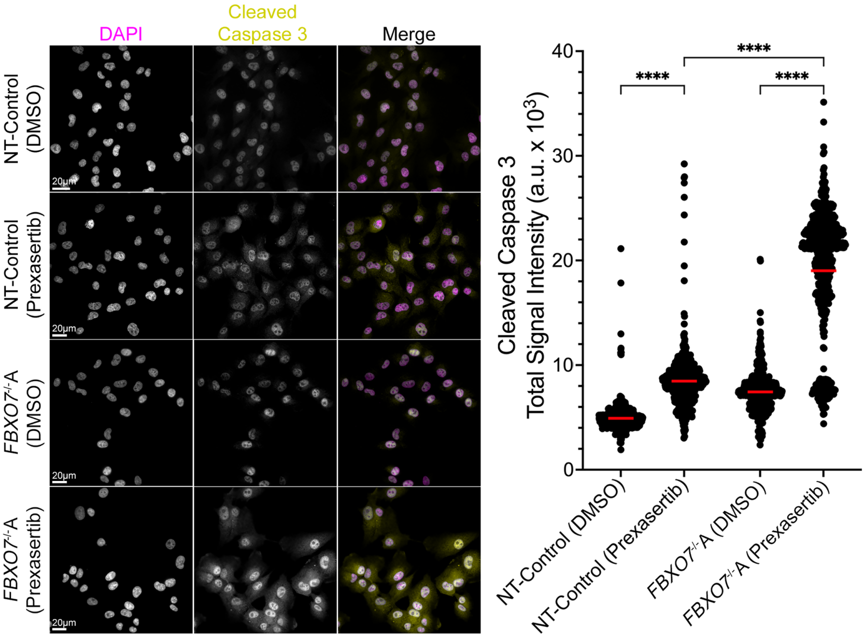

Figure 5. Prexasertib induces preferential increases in cleaved caspase-3 signal intensities in *FBXO7*$^{-/-}$A cells

Prexasertib induces visual (left) and statistical (right) increases in cleaved caspase-3 total signal intensities (i.e., apoptosis) within *FBXO7*$^{-/-}$A cells relative to controls (Table S7). Kruskal-Wallis test with Dunn's multiple comparison post-test (****p value <0.0001), with red lines identifying mean values (n = 1; >550 cells/condition; Table S8).

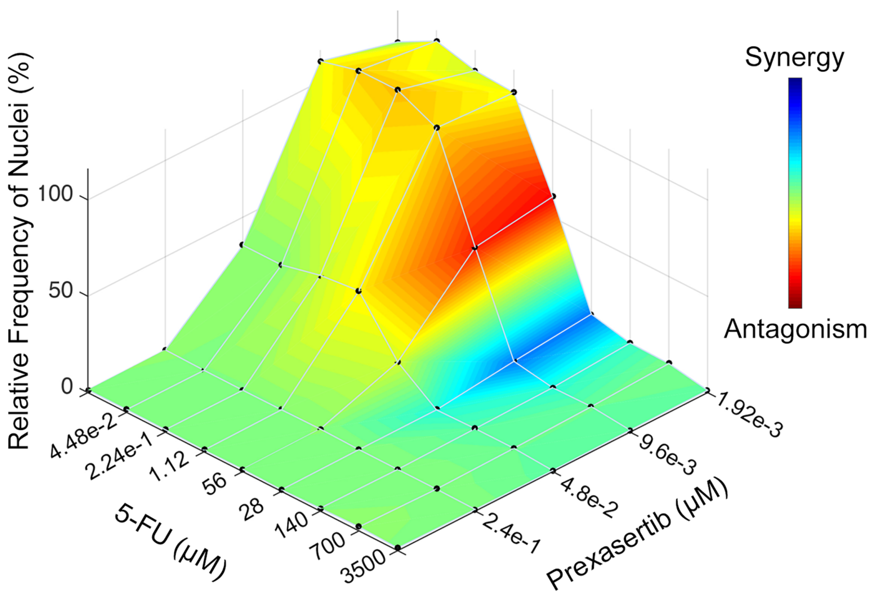

Figure 6. Prexasertib synergizes with 5-FU in *FBXO7*$^{-/-}$A cells

Loewe Additivity model presenting the relative frequency of nuclei remaining. Combenefit analysis reveals a synergistic interaction between Prexasertib and 5-FU within *FBXO7*$^{-/-}$A cells. The lookup table (right) presents the type of interactions ranging from antagonistic (red) to synerigistic (blue). Note the synergistic (blue) interaction occurs between 1.92 nM–9.6 nM Prexasertib and 28 μM 5-FU.

SCF-independent functions, with significant associations to cancer development and progression.[46] One crucial function of FBXO7 is its role as a scaffold protein, stabilizing important cell-cycle regulators such as P27, a cyclin-dependent kinase inhibitor that modulates cell-cycle progression by inhibiting Cyclin E-CDK2 complexes. P27 is a member of the cyclin-dependent kinase inhibitor (CDI) protein family and plays a key role in regulating the G0/G1 to S-phase transition.[47] Previous studies have shown that *FBXO7* deficiency in T cells leads to decreased P27 abundance and promotes cellular proliferation.[48] Importantly, reduced P27 levels are also associated with increased sensitivity to CHEK1 inhibition.[49] This observation suggests that the *FBXO7*:*CHEK1* SL interaction we identified may result from the combined effects of reduced P27 abundance and enhanced sensitivity to CHEK1 inhibition. Given that dysregulation of the cell cycle is a hallmark of cancer,[50] the pathway involving FBXO7, P27, and CHEK1 may represent an exploitable therapeutic target, particularly for cancers exhibiting *FBXO7* copy number losses. The disruption of these interconnected pathways likely induces genome instability and defects in essential cellular processes, potentially accounting for the SL interaction observed in our study. This mechanism underscores the potential of targeting parallel or similar biological pathways to achieve selective cancer cell killing using an SL paradigm.

The *FBXO7*:*CHEK1* SL interaction, while significant, may be modulated by functional redundancy among F-box proteins. FBXO7 is one of 69 F-box proteins in humans, and these proteins frequently exhibit overlapping target substrates and functions, a phenomenon well-documented in plants[51] and likely conserved in humans due to the evolutionarily conserved nature of the SCF complex. This substrate targeting redundancy could potentially compensate for *FBXO7* loss, as the SCF complex regulated numerous proteins, with each F-box protein targeting multiple substrates, some unique and some shared.[22] While these findings are promising, our work focuses exclusively on *FBXO7*:*CHEK1* SL interaction and does not address potential compensatory mechanisms by other F-box proteins, which represent an important avenue for future research. Despite this redundancy potentially influencing the strength of the observed SL phenotype, our findings remain clinically relevant. The *FBXO7*:*CHEK1* interaction could offer therapeutic advantages by potentially reducing the risk of severe side effects typically associated with traditional chemotherapeutics. Moreover, it may prove valuable in enhancing current standard-of-care treatments for CRCs with *FBXO7* deficiencies; however, these possibilities will require further pre-clinical study. Nevertheless, this discovery opens new avenues for targeted cancer therapies. While further research is needed to fully elucidate the mechanisms and potential applications of this SL interaction, our findings provide a promising foundation for developing more effective and personalized treatment strategies in CRC.

Recognizing the challenge of drug resistance in cancers characterized by high levels of CIN,[17] we investigated multi-agent drug sensitivity in an *FBXO7*-deficient model. Our study identified a synergistic relationship between CHEK1 inhibition and 5-FU, which may be attributed to two key mechanisms. First, 5-FU induces replication stress and activates the ATR/CHEK1 pathway.[52] In *FBXO7*-deficient cells, the inability to repair DNA damage associated with the replication stress[11] likely leads to increased genome instability, rendering these cells reliant on CHEK1 for checkpoint activation and survival. This mechanism parallels the *BRCA1/2*:*PARP1* SL interaction that induces DNA single-strand breaks that are not efficiently repaired in cells with DSB repair defects,[26,27] where the *FBXO7*:*CHEK1* interaction enhances replication stress, resulting in increased DSBs and apoptotic death in cells lacking an effective CHEK1-driven checkpoint. Second, our A1309 cellular model expresses a truncated form of adenomatous polyposis coli (APC),[53] which has been implicated as a potential biomarker of 5-FU resistance. Previous studies suggest that CHEK1 inhibition may help overcome this resistance.[52] Consequently, selective inhibition of cell-cycle checkpoints and the DDR with Prexasertib enhances 5-FU sensitivity and induces synergistic killing in *FBXO7*-deficient cells. Overall, our findings indicate that the combination of Prexasertib and 5-FU resensitized

FBXO7-deficient cells to 5-FU treatment, although this effect is observed within a specific concentration range and only within an *in vitro* context. To strengthen the translational relevance of these findings, future studies should incorporate *in vivo* validation using xenotransplant or patient-derived xenograft (PDX) models. These models, which closely mimic the histological and molecular characteristics of human tumors, would allow us to evaluate the therapeutic synergy of Prexasertib and 5-FU under conditions that better replicate the tumor microenvironment. By employing appropriate controls and immunocompromised mouse strains, such as NSG mice, we can further validate the mechanistic insights and therapeutic potential observed *in vitro*. In any case, our findings highlight the potential of targeting multiple pathways to combat drug resistance in CIN cancers and emphasize the importance of precise dosing in combination therapies.

In conclusion, this study provides a novel framework for therapeutically exploiting alterations in SCF complex members, particularly F-box proteins, in CRC. We identified an SL interaction between *FBXO7* and *CHEK1*, demonstrating that CHEK1 inhibition via siRNA silencing or Prexasertib preferentially decreases cell numbers in *FBXO7*-deficient cells. Mechanistic insight revealed increased DSBs and apoptosis following Prexasertib treatment, with a synergistic interaction observed between Prexasertib and 5-FU. Additional, further pre-clinical studies, such as mouse and PDX models will be required to fully elucidate the therapeutic potential of this approach, particularly in combination with current standard-of-care treatments. Additionally, as *FBXO7* copy number losses occur in multiple cancer types, these findings may have broad-spectrum relevance beyond CRC. From a clinical perspective, *FBXO7* loss in patients could be detected using DNA sequencing or array-based comparative genomic hybridization for copy number analysis, quantitative PCR, or RNA sequencing for transcript levels, and immunohistochemistry for protein expression. These tests can be applied to tumor samples and compared with matched normal tissue or blood to confirm somatic alterations and identify potentially responsive patient cohorts. Further investigation into the role of F-box proteins in other cancers could reveal new therapeutic targets and strategies for personalized medicine. Finally, by expanding our understanding of the complex interplay between F-box proteins and cancer progression, we pave the way for innovative approaches aimed at enhancing cancer cell killing, overcoming drug resistance and improving patient outcomes.

MATERIALS AND METHODS

Bioinformatic and statistical approaches

FBXO7 copy number losses data were extracted from TCGA Pan-Cancer Atlas[23] using cBioPortal (www.cbioportal.org).[24] Copy number losses from 10 solid tumor types (bladder, breast, CRC, glioblastoma multiforme [GBM], head and neck, kidney, liver, lung, pancreatic, and prostate) were identified using OncoQuery Language commands: (1) HOMDEL (deep deletion; loss of both alleles), and (2) HETLOSS (shallow deletion; loss of one allele). In general, statistical analyses involving a single comparison (e.g., between two groups), *p* values were reported. For analyses involving multiple comparisons or high-throughput data, q values (i.e., false discovery rate [FDR]; FDR-adjusted *p* values) were calculated using the Benjamini-Hochberg procedure to control for the FDR. In all cases, the type of value reported (*p* or q) is indicated in the figure legends and corresponding supplementary tables. *FBXO7* mRNA expression data from CRC patients were imported into Prism v10 (GraphPad), where cases with shallow deletions were statistically compared with diploid controls using a Mann-Whitney test. Wilcoxon tests with Benjamini-Hochberg FDR corrections were automatically generated in cBioPortal to statistically compare CRC cases with *FBXO7* shallow deletions to unaltered/diploid cases for the fraction of the genome altered, aneuploidy scores, and tumor break load, with a q value <0.05 considered statistically significant. All graphs were imported into Photoshop 2025 (Adobe) where figures were generated. Patient survival, including overall, progression free, and disease-specific, were assessed in cBioPortal[24] using Pan-Cancer Atlas data.[23] Survival data were imported into Prism, where Kaplan-Meier (KM) plots were generated and log rank tests were performed, with a *p* value of <0.05 considered statistically significant. KM plots were exported as TIF images and imported into Photoshop, where figures were assembled.

Cell culture and passaging

Parental A1309[53] cells were generously provided by Dr. Jerry Shay (University of Texas Southwestern Medical Center, TX) and are a non-malignant/non-transformed, human colonic epithelial cell line immortalized with hTERT and CDK4 that also harbor the following CRC-relevant genetic edits: (1) reduced *TP53* expression (short hairpin RNA [shRNA]); (2) mutant $KRAS^{G12V}$ expression; and (3) expression of altered APC (adenomatous polyposis coli) truncated at amino acid 1309. We previously generated two distinct $FBXO7^{-/-}$ clones ($FBXO7^{-/-}$A and $FBXO7^{-/-}$B) along with an NT-Control (contains non-targeting single guide RNA [sgRNA]) clone using a CRISPR-Cas9 approach, which were validated with western blots and DNA sequencing.[11] Parental A1309 cells and the derivative NT-Control clone are karyotypically stable (i.e., do not exhibit CIN), whereas both $FBXO7^{-/-}$ clones are karyotypically unstable and exhibit CIN.[11] All cells were cultured in X-medium (Dulbecco's Modified Eagle's Medium [DMEM] with High Glucose/Medium 199; HyClone) supplemented with 2% cosmic calf serum (CCS; HyClone). Cells were grown in low-oxygen chambers containing 2% O_2, 7% CO_2, and 91% N_2 at 37°C and were authenticated based on protein expression and karyotypic analyses.

siRNA-based gene silencing and western blot

Gene silencing was performed by transfecting ON-TARGETplus (Dharmacon) siRNA duplexes into cells using RNAiMax Transfection Reagent (Life Technologies). Using this approach, we typically attain ≥90% transfection efficiencies as reflected by substantial reductions in protein abundance within the experimental conditions (typically to <10% of endogenous protein levels) relative to the non-targeting siRNA control (siControl). Briefly, four individual siRNA duplexes (e.g., siCHEK1-1, -2, -3, and -4) targeting distinct

coding regions, a pool composed of equimolar concentrations of each individual siRNA (e.g., siCHEK1-P) or siControl, were employed throughout this study. Silencing efficiencies were assessed 4 days post-transfection using standard western blotting techniques as described,[54] with the following primary antibodies targeting FBXO7 (Abcam; ab154098; 1:5,000), CHEK1 (Abcam; ab40866; 1:5,000) and Cyclophilin B (loading control; Abcam; ab16045; 1:50,000), and secondary antibodies (Goat anti-Rabbit horseradish peroxidase [HRP] [Jackson ImmunoResearch; l11-035-144; 1:15,000] and Goat anti-Mouse HRP [Jackson ImmunoResearch; 115-035-146; 1:10,000]). Semi-quantitative western blots were conducted as detailed,[40] with silencing efficiencies assessed using ImageJ (Gel Analyzer tool). Briefly, band intensities were first normalized to the corresponding Cyclophilin B loading control and are presented relative to the siControl band, which is set to 100%.

QuantIM analyses of cell numbers

Imaging of 96-well plates was performed to quantify cellular numbers following silencing or drug treatments using a Cytation3 Cell Imaging Multi-Mode Reader (BioTek), equipped with a 16-bit charge-coupled device camera (Sony). For each well, 16 non-overlapping images (4 × 4 matrix) were acquired using an Olympus 10× objective lens (0.3 numerical aperture). Exposure times for the Hoechst channel were optimized and Gen5 software was used to automatically quantify the number of nuclei (surrogate marker of cell numbers) per well. To account for potential differences in growth rates between the NT-Control and the *FBXO7*$^{-/-}$ clones, nuclear counts from each well were normalized to the average number of nuclear counts for the control conditions (i.e., siControl or DMSO for the siRNA or drug treatment experiments, respectively). All nuclear count data were imported into Prism, where the average nuclear counts from six replicates were determined and normalized to the average nuclear count from the respective control. Normalized nuclear counts from the NT-Control were statistically compared with the normalized nuclear counts from each experimental condition in the *FBXO7*$^{-/-}$ clones using multiple paired t tests, with the two-stage step-up multi-comparison correction method by Benjamini, Krieger, and Yekutieli (FDR; Q = 5%) in Prism with a q value <0.05 considered significant. Graphs were generated in Prism and exported into Photoshop 2025, where figures were assembled.

Prexasertib dose-response curves

Dose-response curves were generated as detailed elsewhere[40] with slight modifications as detailed below. Briefly, NT-Control, *FBXO7*$^{-/-}$A, and *FBXO7*$^{-/-}$B clones were seeded into 96-well plates, allowed to attach and grow for 24 h prior to treatment with 5-fold serial dilution series (15.4 pM to 6 μM) of Prexasertib (RayBiotech; 332–11821), a selective CHEK1 inhibitor, or vehicle control (DMSO), with all conditions performed in sextuplet. Cells were permitted to grow for 4 days (approximately four population doublings), at which point they were fixed (4% paraformaldehyde), counterstained (Hoechst 33342), and subjected to QuantIM (detailed below). In brief, nuclear counts (surrogate marker for cell numbers) from each condition were extracted from image series collected for each cell line and drug concentration and were normalized to the respective DMSO-treated controls. Relative cell numbers were imported into Prism, where standard dose-response curves were generated, and the effective concentration at 50% (EC_{50}) values determined. The concentration of Prexasertib (6.0 nM) inducing the greatest SL phenotype within *FBXO7*$^{-/-}$ clones was assessed in subsequent direct tests and employed in all subsequent work.

QuantIM assessments of DNA DSBs and apoptosis

Asynchronous NT-Control and *FBXO7*$^{-/-}$A were seeded onto sterilized coverslips, allowed to attach and begin growing for 24 h, at which point they were treated and incubated with Prexasertib (6.0 nM) or DMSO for 48 h. Following the 48 h incubation period, cells were fixed (4% paraformaldehyde), permeabilized (0.5% Triton X-100 in PBS; 10 min), and immunofluorescently labeled for 1 h with either anti-γ-H2AX (Abcam; ab26350; 1:200) or anti-cleaved caspase-3 (Abcam; ab13847; 1:200) antibodies followed by 1 h incubations with goat anti-mouse-Cy3 (Abcam; ab97035; 1:200) or goat-anti-rabbit-Alexa Fluor 488 (Abcam; ab150081; 1:200) secondary antibodies, respectively. Coverslips were mounted onto slides with Vectashield containing DAPI to counterstain nuclei and subjected to QuantIM as described.[55] Briefly, each channel was independently optimized, and exposure times were maintained constant throughout the acquisition phase (Zeiss Axio Imager 2; 20× objective). Image analyses quantified either the total number of γ-H2AX foci/cell, or the cleaved caspase-3 total signal intensity for each interphase cell imaged, with a minimum of 550 cells imaged/condition. Kruskal-Wallis tests with Dunn's multiple comparison post-tests statistically compare the total number of γ-H2AX foci or cleaved caspase-3 total signal intensities with DMSO-treated controls, with a p value of <0.05 considered significant. Descriptive statistics (N; mean ± standard deviation [SD]) and scatterplots were generated in Prism and imported into Photoshop 2025, where figures were assembled.

Combinatorial dose-response curves

NT-Control and *FBXO7*$^{-/-}$A clones were seeded into 96-well plates, permitted to attach and grow for 24 h prior to treatment with DMSO, or varying concentrations of Prexasertib (0.384, 1.92 nM, 9.6 nM, 48 nM, and 240 nM), 5-fluorouracil (5-FU; 8.96 nM, 44.8 nM, 224 nM, 1.12 μM, 5.6 μM, 28 μM, 140 μM, and 700 μM), or both Prexasertib and 5-FU. Cells were permitted to grow for 4 additional days, at which point they were fixed, counterstained, imaged, and analyzed as above with all nuclear counts normalized to the corresponding DMSO control for the respective cell line. Cell counts were imported into Combenefit software,[45] where they were assessed by the Loewe Additivity model for antagonistic or synergistic drug interactions.

DATA AVAILABILITY

All authors confirm that all supporting data and figures for this study can be found in the article or the associated supplemental information.

ACKNOWLEDGMENTS

No ethics approval was required to conduct these studies due to the use of publicly available datasets from TCGA Research Network (https://www.cancer.gov/tcga)[23] and were accessed online through cBioPortal[24] from Nov 21–29, 2024. No other human specimens or clinical data were utilized. We acknowledge that the University of Manitoba and the Paul Albrechtsen Research Institute are located on the original lands of Anishinaabeg, Ininiwak, Anisininewuk, Dakota Oyate and Dene, and on the National homeland of the Red River Métis. We respect the Treaties that were made on these territories and acknowledge the harms and mistakes of the past. We dedicate ourselves to move forward in partnership with Indigenous communities in a spirit of reconciliation and collaboration. We thank members of the McManus laboratory for constructive criticism during the writing of this manuscript. We also thank Dr. Jerry Shay for generously providing the parental A1309 cell line and acknowledge the strong support of the Paul Albrechtsen Research Institute and the Quantitative Imaging, Phenotyping and Sorting (QuIPS) Platform (QuIPSPlatform.ca) supported by the CancerCare Manitoba Foundation. Research in the McManus laboratory was generously supported by a Max Rady College of Medicine, BSc Medicine Studentship (T.R.), a Canadian Institutes of Health Research Project Grant (162374; K.J.M.), and a CancerCare Manitoba Foundation Operating Grant (K.J.M.).

AUTHOR CONTRIBUTIONS

T.R.: Conceptualization, data curation, formal analyses, investigation, methodology, writing – original draft, and writing – review & editing. A.C.F: Conceptualization, methodology, and supervision. R.C.G.: Data curation, formal analyses, investigation, methodology, and writing – review & editing. N.M.N.: Data curation, formal analyses, investigation, and methodology. Z.L.: Data curation, formal analyses, investigation and methodology. K.J.M.: Conceptualization, data curation, formal analyses, funding acquisition, investigation, project administration, supervision, writing – original draft, and writing – review & editing.

DECLARATION OF INTERESTS

The authors declare that they have no known competing financial interests or personal relationships that could have appeared to influence the work reported in this paper.

SUPPLEMENTAL INFORMATION

Supplemental information can be found online at https://doi.org/10.1016/j.omton.2025.201028.

REFERENCES

1. Bray, F., Laversanne, M., Sung, H., Ferlay, J., Siegel, R.L., Soerjomataram, I., and Jemal, A. (2024). Global cancer statistics 2022: GLOBOCAN estimates of incidence and mortality worldwide for 36 cancers in 185 countries. CA Cancer J. Clin. *74*, 229–263. https://doi.org/10.3322/caac.21834.

2. Morgan, E., Arnold, M., Gini, A., Lorenzoni, V., Cabasag, C.J., Laversanne, M., Vignat, J., Ferlay, J., Murphy, N., and Bray, F. (2023). Global burden of colorectal cancer in 2020 and 2040: incidence and mortality estimates from GLOBOCAN. Gut *72*, 338–344. https://doi.org/10.1136/gutjnl-2022-327736.

3. Lopes, S.R., Martins, C., Santos, I.C., Teixeira, M., Gamito, É., and Alves, A.L. (2024). Colorectal cancer screening: A review of current knowledge and progress in research. World J. Gastrointest. Oncol. *16*, 1119–1133. https://doi.org/10.4251/wjgo.v16.i4.1119.

4. Siegel, R.L., Wagle, N.S., Cercek, A., Smith, R.A., and Jemal, A. (2023). Colorectal cancer statistics, 2023. CA Cancer J. Clin. *73*, 233–254. https://doi.org/10.3322/caac.21772.

5. Lengauer, C., Kinzler, K.W., and Vogelstein, B. (1997). Genetic instability in colorectal cancers. Nature *386*, 623–627. https://doi.org/10.1038/386623a0.

6. Geigl, J.B., Obenauf, A.C., Schwarzbraun, T., and Speicher, M.R. (2008). Defining 'chromosomal instability. Trends Genet. *24*, 64–69. https://doi.org/10.1016/j.tig.2007.11.006.

7. Vishwakarma, R., and McManus, K.J. (2020). Chromosome Instability; Implications in Cancer Development, Progression, and Clinical Outcomes. Cancers (Basel) *12*, 824. https://doi.org/10.3390/cancers12040824.

8. Campos Gudino, R., Neudorf, N.M., Andromidas, D., Lichtensztejn, Z., and McManus, K.J. (2024). Loss of EMI1 compromises chromosome stability and is associated with cellular transformation in colonic epithelial cell contexts. Br. J. Cancer *131*, 1516–1528. https://doi.org/10.1038/s41416-024-02855-9.

9. Lepage, C.C., Palmer, M.C.L., Farrell, A.C., Neudorf, N.M., Lichtensztejn, Z., Nachtigal, M.W., and McManus, K.J. (2021). Reduced SKP1 and CUL1 expression underlies increases in Cyclin E1 and chromosome instability in cellular precursors of high-grade serous ovarian cancer. Br. J. Cancer *124*, 1699–1710. https://doi.org/10.1038/s41416-021-01317-w.

10. Neudorf, N.M., Thompson, L.L., Lichtensztejn, Z., Razi, T., and McManus, K.J. (2022). Reduced SKP2 Expression Adversely Impacts Genome Stability and Promotes Cellular Transformation in Colonic Epithelial Cells. Cells *11*, 3731. https://doi.org/10.3390/cells11233731.

11. Palmer, M.C.L., Neudorf, N.M., Farrell, A.C., Razi, T., Lichtensztejn, Z., and McManus, K.J. (2022). The F-box protein, FBXO7, is required to maintain chromosome stability in humans. Hum. Mol. Genet. *31*, 1471–1486. https://doi.org/10.1093/hmg/ddab330.

12. Bungsy, M., Palmer, M.C.L., Jeusset, L.M., Neudorf, N.M., Lichtensztejn, Z., Nachtigal, M.W., and McManus, K.J. (2021). Reduced RBX1 expression induces chromosome instability and promotes cellular transformation in high-grade serous ovarian cancer precursor cells. Cancer Lett. *500*, 194–207. https://doi.org/10.1016/j.canlet.2020.11.051.

13. Janiszewska, M. (2020). The microcosmos of intratumor heterogeneity: the space-time of cancer evolution. Oncogene *39*, 2031–2039. https://doi.org/10.1038/s41388-019-1127-5.

14. Oh, B.Y., Shin, H.T., Yun, J.W., Kim, K.T., Kim, J., Bae, J.S., Cho, Y.B., Lee, W.Y., Yun, S.H., Park, Y.A., et al. (2019). Intratumor heterogeneity inferred from targeted deep sequencing as a prognostic indicator. Sci. Rep. *9*, 4542. https://doi.org/10.1038/s41598-019-41098-0.

15. Bakhoum, S.F., Ngo, B., Laughney, A.M., Cavallo, J.A., Murphy, C.J., Ly, P., Shah, P., Sriram, R.K., Watkins, T.B.K., Taunk, N.K., et al. (2018). Chromosomal instability drives metastasis through a cytosolic DNA response. Nature *553*, 467–472. https://doi.org/10.1038/nature25432.

16. Gao, C., Su, Y., Koeman, J., Haak, E., Dykema, K., Essenberg, C., Hudson, E., Petillo, D., Khoo, S.K., and Vande Woude, G.F. (2016). Chromosome instability drives phenotypic switching to metastasis. Proc. Natl. Acad. Sci. USA *113*, 14793–14798. https://doi.org/10.1073/pnas.1618215113.

17. Lee, A.J.X., Endesfelder, D., Rowan, A.J., Walther, A., Birkbak, N.J., Futreal, P.A., Downward, J., Szallasi, Z., Tomlinson, I.P.M., Howell, M., et al. (2011). Chromosomal instability confers intrinsic multidrug resistance. Cancer Res. *71*, 1858–1870. https://doi.org/10.1158/0008-5472.CAN-10-3604.

18. Wang, W., Zhang, Y., Chen, R., Tian, Z., Zhai, Y., Janz, S., Gu, C., and Yang, Y. (2017). Chromosomal instability and acquired drug resistance in multiple myeloma. Oncotarget *8*, 78234–78244. https://doi.org/10.18632/oncotarget.20829.

19. Choi, C.M., Seo, K.W., Jang, S.J., Oh, Y.M., Shim, T.S., Kim, W.S., Lee, D.S., and Lee, S.D. (2009). Chromosomal instability is a risk factor for poor prognosis of adenocarcinoma of the lung: Fluorescence in situ hybridization analysis of paraffin-embedded tissue from Korean patients. Lung Cancer *64*, 66–70. https://doi.org/10.1016/j.lungcan.2008.07.016.

20. Kikutake, C., Yoshihara, M., Sato, T., Saito, D., and Suyama, M. (2018). Pan-cancer analysis of intratumor heterogeneity associated with patient prognosis using multidimensional measures. Oncotarget *9*, 37689–37699. https://doi.org/10.18632/oncotarget.26485.

21. Walther, A., Houlston, R., and Tomlinson, I. (2008). Association between chromosomal instability and prognosis in colorectal cancer: a meta-analysis. Gut *57*, 941–950. https://doi.org/10.1136/gut.2007.135004.

22. Lee, E.K., and Diehl, J.A. (2014). SCFs in the new millennium. Oncogene *33*, 2011–2018. https://doi.org/10.1038/onc.2013.144.

23. Hoadley, K.A., Yau, C., Hinoue, T., Wolf, D.M., Lazar, A.J., Drill, E., Shen, R., Taylor, A.M., Cherniack, A.D., Thorsson, V., et al. (2018). Cell-of-Origin Patterns Dominate the Molecular Classification of 10,000 Tumors from 33 Types of Cancer. Cell *173*, 291–304.e6. https://doi.org/10.1016/j.cell.2018.03.022.

24. Gao, J., Aksoy, B.A., Dogrusoz, U., Dresdner, G., Gross, B., Sumer, S.O., Sun, Y., Jacobsen, A., Sinha, R., Larsson, E., et al. (2013). Integrative analysis of complex

cancer genomics and clinical profiles using the cBioPortal. Sci. Signal. *6*, pl1. https://doi.org/10.1126/scisignal.2004088.

25. O'Neil, N.J., Bailey, M.L., and Hieter, P. (2017). Synthetic lethality and cancer. Nat. Rev. Genet. *18*, 613–623. https://doi.org/10.1038/nrg.2017.47.
26. Bryant, H.E., Schultz, N., Thomas, H.D., Parker, K.M., Flower, D., Lopez, E., Kyle, S., Meuth, M., Curtin, N.J., and Helleday, T. (2005). Specific killing of BRCA2-deficient tumours with inhibitors of poly(ADP-ribose) polymerase. Nature *434*, 913–917. https://doi.org/10.1038/nature03443.
27. Farmer, H., McCabe, N., Lord, C.J., Tutt, A.N.J., Johnson, D.A., Richardson, T.B., Santarosa, M., Dillon, K.J., Hickson, I., Knights, C., et al. (2005). Targeting the DNA repair defect in BRCA mutant cells as a therapeutic strategy. Nature *434*, 917–921. https://doi.org/10.1038/nature03445.
28. Dai, Y., and Grant, S. (2010). New insights into checkpoint kinase 1 in the DNA damage response signaling network. Clin. Cancer Res. *16*, 376–383. https://doi.org/10.1158/1078-0432.CCR-09-1029.
29. Zhang, Y.W., Otterness, D.M., Chiang, G.G., Xie, W., Liu, Y.C., Mercurio, F., and Abraham, R.T. (2005). Genotoxic stress targets human Chk1 for degradation by the ubiquitin-proteasome pathway. Mol. Cell *19*, 607–618. https://doi.org/10.1016/j.molcel.2005.07.019.
30. Zhou, J., Sanchez-Vega, F., Caso, R., Tan, K.S., Brandt, W.S., Jones, G.D., Yan, S., Adusumilli, P.S., Bott, M., Huang, J., et al. (2019). Analysis of Tumor Genomic Pathway Alterations Using Broad-Panel Next-Generation Sequencing in Surgically Resected Lung Adenocarcinoma. Clin. Cancer Res. *25*, 7475–7484. https://doi.org/10.1158/1078-0432.CCR-19-1651.
31. Taylor, A.M., Shih, J., Ha, G., Gao, G.F., Zhang, X., Berger, A.C., Schumacher, S.E., Wang, C., Hu, H., Liu, J., et al. (2018). Genomic and Functional Approaches to Understanding Cancer Aneuploidy. Cancer Cell *33*, 676–689.e3. https://doi.org/10.1016/j.ccell.2018.03.007.
32. Lakbir, S., Lahoz, S., Cuatrecasas, M., Camps, J., Glas, R.A., Heringa, J., Meijer, G.A., Abeln, S., and Fijneman, R.J.A. (2022). Tumour break load is a biologically relevant feature of genomic instability with prognostic value in colorectal cancer. Eur. J. Cancer *177*, 94–102. https://doi.org/10.1016/j.ejca.2022.09.034.
33. Guppy, B.J., and McManus, K.J. (2017). Synthetic lethal targeting of RNF20 through PARP1 silencing and inhibition. Cell. Oncol. *40*, 281–292. https://doi.org/10.1007/s13402-017-0323-y.
34. McAndrew, E.N., Lepage, C.C., and McManus, K.J. (2016). The synthetic lethal killing of RAD54B-deficient colorectal cancer cells by PARP1 inhibition is enhanced with SOD1 inhibition. Oncotarget *7*, 87417–87430. https://doi.org/10.18632/oncotarget.13654.
35. Sajesh, B.V., and McManus, K.J. (2015). Targeting SOD1 induces synthetic lethal killing in BLM- and CHEK2-deficient colorectal cancer cells. Oncotarget *6*, 27907–27922. https://doi.org/10.18632/oncotarget.4875.
36. McManus, K.J., Barrett, I.J., Nouhi, Y., and Hieter, P. (2009). Specific synthetic lethal killing of RAD54B-deficient human colorectal cancer cells by FEN1 silencing. Proc. Natl. Acad. Sci. USA *106*, 3276–3281. https://doi.org/10.1073/pnas.0813414106.
37. Jeusset, L.M., and McManus, K.J. (2021). Exploring Candidate Human Synthetic Lethal Interactions Through siRNA and Quantitative Imaging-Based Approaches. Methods Mol. Biol. *2381*, 151–173. https://doi.org/10.1007/978-1-0716-1740-3_8.
38. Wu, X., Seraia, E., Hatch, S.B., Wan, X., Ebner, D.V., Aroldi, F., Jiang, Y., Ryan, A.J., Bogenrieder, T., Weyer-Czernilofsky, U., et al. (2022). CHK1 inhibition exacerbates replication stress induced by IGF blockade. Oncogene *41*, 476–488. https://doi.org/10.1038/s41388-021-02080-1.
39. Tsang, C.K., Liu, Y., Thomas, J., Zhang, Y., and Zheng, X.F.S. (2014). Superoxide dismutase 1 acts as a nuclear transcription factor to regulate oxidative stress resistance. Nat. Commun. *5*, 3446. https://doi.org/10.1038/ncomms4446.
40. Sajesh, B.V., Bailey, M., Lichtensztejn, Z., Hieter, P., and McManus, K.J. (2013). Synthetic lethal targeting of superoxide dismutase 1 selectively kills RAD54B-deficient colorectal cancer cells. Genetics *195*, 757–767. https://doi.org/10.1534/genetics.113.156836.
41. Porter, A.G., and Jänicke, R.U. (1999). Emerging roles of caspase-3 in apoptosis. Cell Death Differ. *6*, 99–104. https://doi.org/10.1038/sj.cdd.4400476.
42. Ghafouri-Fard, S., Abak, A., Tondro Anamag, F., Shoorei, H., Fattahi, F., Javadinia, S.A., Basiri, A., and Taheri, M. (2021). 5-Fluorouracil: A Narrative Review on the Role of Regulatory Mechanisms in Driving Resistance to This Chemotherapeutic Agent. Front. Oncol. *11*, 658636. https://doi.org/10.3389/fonc.2021.658636.
43. Suetsugu, T., Mori, R., Futamura, M., Fukada, M., Tanaka, H., Yasufuku, I., Sato, Y., Iwata, Y., Imai, T., Imai, H., et al. (2021). Mechanism of acquired 5FU resistance and strategy for overcoming 5FU resistance focusing on 5FU metabolism in colon cancer cell lines. Oncol. Rep. *45*, 27. https://doi.org/10.3892/or.2021.7978.
44. Zhang, N., Yin, Y., Xu, S.J., and Chen, W.S. (2008). 5-Fluorouracil: mechanisms of resistance and reversal strategies. Molecules *13*, 1551–1569. https://doi.org/10.3390/molecules13081551.
45. Di Veroli, G.Y., Fornari, C., Wang, D., Mollard, S., Bramhall, J.L., Richards, F.M., and Jodrell, D.I. (2016). Combenefit: an interactive platform for the analysis and visualization of drug combinations. Bioinformatics *32*, 2866–2868. https://doi.org/10.1093/bioinformatics/btw230.
46. Nelson, D.E., Randle, S.J., and Laman, H. (2013). Beyond ubiquitination: the atypical functions of Fbxo7 and other F-box proteins. Open Biol. *3*, 130131. https://doi.org/10.1098/rsob.130131.
47. Chu, I.M., Hengst, L., and Slingerland, J.M. (2008). The Cdk inhibitor p27 in human cancer: prognostic potential and relevance to anticancer therapy. Nat. Rev. Cancer *8*, 253–267. https://doi.org/10.1038/nrc2347.
48. Patel, S.P., Randle, S.J., Gibbs, S., Cooke, A., and Laman, H. (2017). Opposing effects on the cell cycle of T lymphocytes by Fbxo7 via Cdk6 and p27. Cell. Mol. Life Sci. *74*, 1553–1566. https://doi.org/10.1007/s00018-016-2427-3.
49. Lohmuller, M., Roeck, B.F., Szabo, T.G., Schapfl, M.A., Pegka, F., Herzog, S., Villunger, A., and Schuler, F. (2022). The SKP2-p27 axis defines susceptibility to cell death upon CHK1 inhibition. Mol. Oncol. *16*, 2771–2787. https://doi.org/10.1002/1878-0261.13264.
50. Hanahan, D. (2022). Hallmarks of Cancer: New Dimensions. Cancer Discov. *12*, 31–46. https://doi.org/10.1158/2159-8290.CD-21-1059.
51. Ikram, S., Durandet, M., Vesa, S., Pereira, S., Guerche, P., and Bonhomme, S. (2014). Functional redundancy and/or ongoing pseudogenization among F-box protein genes expressed in Arabidopsis male gametophyte. Plant Reprod. *27*, 95–107. https://doi.org/10.1007/s00497-014-0243-1.
52. Martino-Echarri, E., Henderson, B.R., and Brocardo, M.G. (2014). Targeting the DNA replication checkpoint by pharmacologic inhibition of Chk1 kinase: a strategy to sensitize APC mutant colon cancer cells to 5-fluorouracil chemotherapy. Oncotarget *5*, 9889–9900. https://doi.org/10.18632/oncotarget.2475.
53. Roig, A.I., Eskiocak, U., Hight, S.K., Kim, S.B., Delgado, O., Souza, R.F., Spechler, S.J., Wright, W.E., and Shay, J.W. (2010). Immortalized epithelial cells derived from human colon biopsies express stem cell markers and differentiate in vitro. Gastroenterology *138*, 1012–1021.e1-5. https://doi.org/10.1053/j.gastro.2009.11.052.
54. Barber, T.D., McManus, K., Yuen, K.W.Y., Reis, M., Parmigiani, G., Shen, D., Barrett, I., Nouhi, Y., Spencer, F., Markowitz, S., et al. (2008). Chromatid cohesion defects may underlie chromosome instability in human colorectal cancers. Proc. Natl. Acad. Sci. USA *105*, 3443–3448. https://doi.org/10.1073/pnas.0712384105.
55. McManus, K.J., and Hendzel, M.J. (2005). ATM-dependent DNA damage-independent mitotic phosphorylation of H2AX in normally growing mammalian cells. Mol. Biol. Cell *16*, 5013–5025. https://doi.org/10.1091/mbc.e05-01-0065.

OMTON, Volume *33*

Supplemental information

CHEK1 is a synthetic lethal interactor of *FBXO7* in colonic epithelial cells

Tooba Razi, Ally C. Farrell, Rubi Campos Gudiño, Nicole M. Neudorf, Zelda Lichtensztejn, and Kirk J. McManus

SUPPLEMENTAL MATERIAL

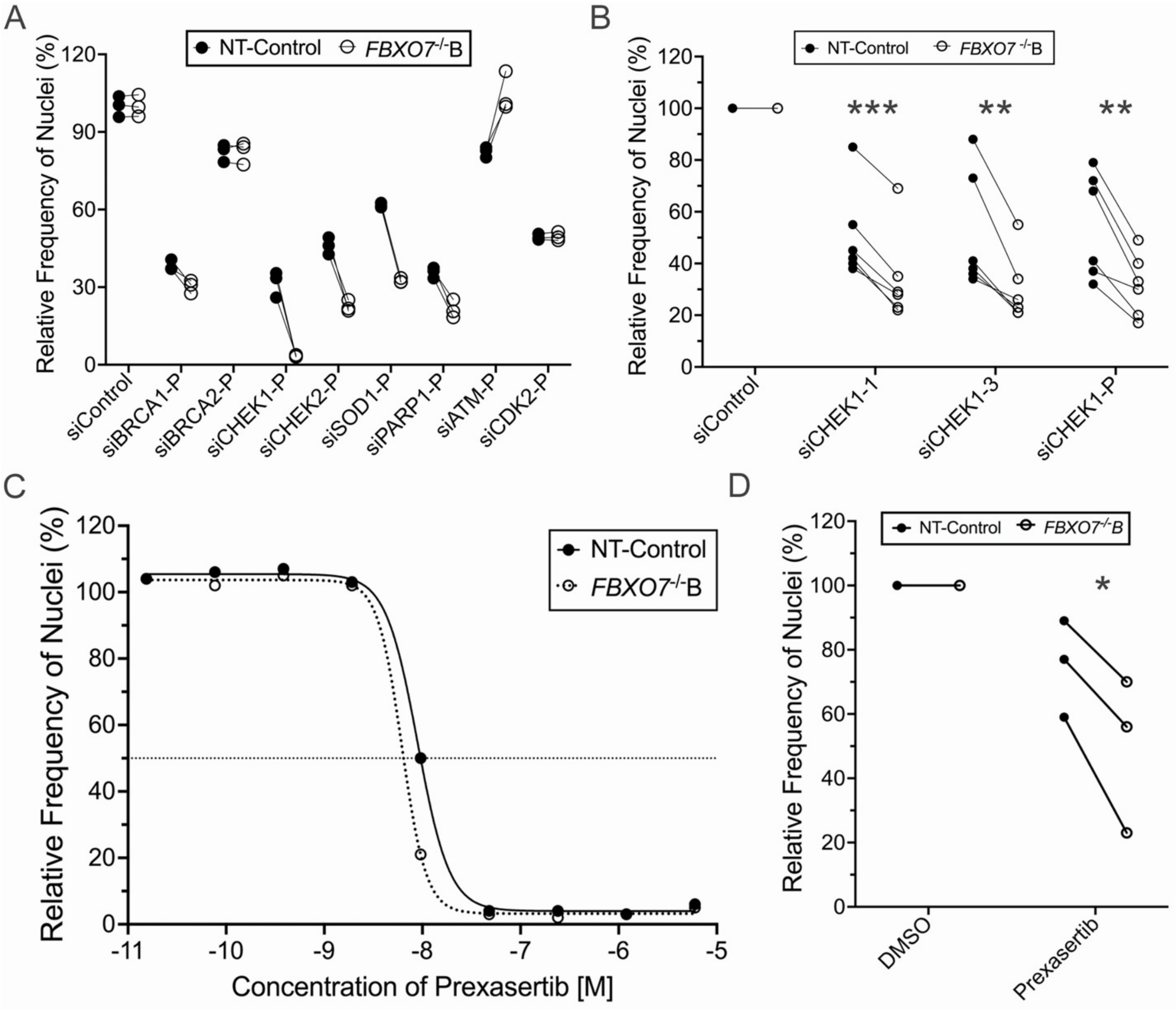

Figure S1: *FBXO7*$^{-/-}$B cells are Hypersensitive to CHEK1 Silencing and Inhibition.
(**A**) Pilot screen reveals *FBXO7*$^{-/-}$B cells are hypersensitive to *CHEK1*, *CHEK2*, *SOD1* and *PARP1* silencing (n = 3). **(B)** QuantIM identifies statistically significant decreases in the relative frequency of *FBXO7*$^{-/-}$B nuclei (cells) relative to NT-Control following *CHEK1* silencing (n = 6, multiple paired t-tests, with Benjamini, Krieger and Yekutieli correction; FDR = 5%; **, q-value < 0.01; ***, q-value < 0.001; Table S2). **(C)** Dose response curve reveals *FBXO7*$^{-/-}$B cells are hypersensitive to Prexasertib treatments (5-fold serial dilution) relative to NT-Control. Mean values are presented normalized to the corresponding DMSO control (n = 6). **(D)** QuantIM identifies a statistically significant decrease in the relative frequency of *FBXO7*$^{-/-}$B nuclei (cells) relative to NT-Control cells following Prexasertib (6.0μM) treatment (n = 3, multiple paired t-tests, with Benjamini, Krieger and Yekutieli correction; FDR = 5%; *, q-value < 0.05; Table S4).

Table S1. ***CHEK1*** **Silencing Induces Statistically Significant Decreases in the Number of** ***FBXO7*** $^{-/-}$**A Cells**[A]**.**

	p-value ($\times 10^{-2}$)	Mean (NT-Cntl)	Mean (*FBXO7* $^{-/-}$)	Diff[B]	SE[C]	t-ratio	df[D]	q-value[E] ($\times 10^{-2}$)
**siControl**		100.0	100.0	0.00	0.00			
**siCHEK1-1**	2.1221	52.7	29.5	23.2	7.00	3.31	5	2.526
**siCHEK1-3**	2.4056	52.5	29.8	22.7	7.09	3.20	5	2.526
**siCHEK1-P**	1.0794	51.2	25.5	25.7	6.49	4.00	5	2.526

[A]Multiple paired t-tests
[B]Difference, Diff
[C]Standard error, SE
[D]Degrees of freedom, df
[E]two-stage step-up Benjamini, Krieger and Yekutieli multiple comparison correction with Q = 5%

Table S2. ***CHEK1*** **Silencing Induces Statistically Significant Decreases in the Number of** ***FBXO7*** $^{-/-}$**B Cells**[A]**.**

	p-value ($\times 10^{-2}$)	Mean (NT-Cntl)	Mean (*FBXO7* $^{-/-}$)	Diff[B]	SE[C]	t-ratio	df[D]	q-value[E] ($\times 10^{-2}$)
**siControl**		100.0	100.0	0.00	0.00			
**siCHEK1-1**	0.0108	50.8	34.33	16.5	1.50	11.0	5	0.0340
**siCHEK1-3**	0.6783	51.7	30.00	21.7	4.88	4.44	5	0.6851
**siCHEK1-P**	0.3384	54.8	31.50	23.3	4.46	2.23	5	0.5127

[A]Multiple paired t-tests
[B]Difference, Diff
[C]Standard error, SE
[D]Degrees of freedom, df
[E]two-stage step-up Benjamini, Krieger and Yekutieli multiple comparison correction with Q = 5%

Table S3. Prexasertib Induces a Significant Decreases in the Number of ***FBXO7*** $^{-/-}$**A Cells.**

**Condition**	p-value ($\times 10^{-2}$)	Mean (NT-Cntl)	Mean (*FBXO7* $^{-/-}$)	Diff[B]	SE[C]	t-ratio	df[D]	q-value[E] ($\times 10^{-2}$)
DMSO		100.0	100.0	0.00	0.00			
Prexasertib	4.0826	72.7	40.33	32.3	6.74	4.80	2	4.286

[A]Multiple paired t-tests
[B]Difference, Diff
[C]Standard error, SE
[D]Degrees of freedom, df
[E]two-stage step-up Benjamini, Krieger and Yekutieli multiple comparison correction with Q = 5%

Table S4. Prexasertib Induces a Significant Decreases in the Number of *FBXO7*$^{-/-}$B Cells.

Condition	p-value (×10^{-2})	Mean (NT-Cntl)	Mean (*FBXO7*$^{-/-}$)	Diff^{B}	SE^{C}	t-ratio	df^{D}	q-value^{E} (×10^{-2})
DMSO		100.0	100.0	0.00	0.00			
Prexasertib	4.2034	75.00	49.7	25.33	5.36	4.72	2	4.414

[A]Multiple paired t-tests
[B]Difference, Diff
[C]Standard error, SE
[D]Degrees of freedom, df
[E]two-stage step-up Benjamini, Krieger and Yekutieli multiple comparison correction with Q = 5%

Table S5. Prexasertib Induces Preferential Increases in γ-H2AX Foci in *FBXO7*$^{-/-}$A Cells.

Cell Type	Treatment	N^{A}	Mean ± SD^{B}	Fold Increase^{C}
NT-Control	DMSO	750	0.2067 ± 1.115	NA
NT-Control	Prexasertib	693	3.221 ± 16.03	15.6
FBXO7$^{-/-}$A	DMSO	596	0.4279 ± 1.462	NA
FBXO7$^{-/-}$A	Prexasertib	552	17.81 ± 36.75	41.6

[A]Number (N)
[B]Standard Deviation (SD)
[C]Relative to the corresponding DMSO-treated control of the identical cell line; Not applicable (NA)

Table S6. Dunn's Multicomparison Tests Reveals Statistically Significant Increases γ-H2AX Foci Following Prexasertib Treatments.

	NT-Control (Prexasertib)	*FBXO7*$^{-/-}$A (DMSO)	*FBOX7*$^{-/-}$A (Prexasertib)
NT-Control (DMSO)	****[A]	****	****
NT-Control (Prexasertib)	****	ns	****
FBXO7$^{-/-}$A (DMSO)			****

[A]Statistical Significance; p-value > 0.05 (ns); p-value <0.0001 (****)

Table S7. Prexasertib Induces Preferential Increases in Cleaved Caspase 3 Total Signal Intensities in *FBXO7*$^{-/-}$A Cells.

Cell Type	Treatment	N[A]	Mean ± SD[B]	Fold Increase[C]
NT-Control	DMSO	799	4922 ± 1249	NA
NT-Control	Prexasertib	611	8474 ± 2690	1.7
FBXO7$^{-/-}$A	DMSO	637	7439 ± 1920	NA
FBXO7$^{-/-}$A	Prexasertib	574	19012 ± 6020	2.6

[A]Number (N)
[B]Standard Deviation (SD)
[C]Relative to the corresponding DMSO-treated control of the identical cell line; Not applicable (NA)

Table S8. Dunn's Multicomparison Tests Reveals Statistically Significant Increases in Cleaved Caspase 3 Total Signal Intensities Following Prexasertib Treatments.

	NT-Control (Prexasertib)	*FBXO7*$^{-/-}$A (DMSO)	*FBOX7*$^{-/-}$A (Prexasertib)
NT-Control (DMSO)	****[A]	****	****
NT-Control (Prexasertib)		****	****
FBXO7$^{-/-}$A (DMSO)			****

[A]Statistical Significance; p-value <0.0001 (****)
